# Is a Transdisciplinary Theory of Engagement in Organized Settings Possible? A Concept Analysis of the Literature on Employee Engagement, Consumer Engagement and Patient Engagement

**DOI:** 10.3389/fpsyg.2017.00872

**Published:** 2017-07-06

**Authors:** Guendalina Graffigna

**Affiliations:** Faculty of Psychology, Università Cattolica del Sacro CuoreMilan, Italy

**Keywords:** engagement, employee engagement, patient engagement, consumer engagement, work engagement, organizational settings, conceptual analysis

## Abstract

Organizations are experiencing increased competition, disruptive innovation, and continuous changes in their social and economic context. Furthermore, the decrease of resources (economic and human) in such a demanding context make it imperative for organizations to find new models and strategies to make their service delivery more sustainable at the economic, environmental and psychological levels. In such a complex scenario the concept of engagement of the individuals involved in organized settings (either as service providers or as final receivers) is a promising lever for innovation. However, despite the number of studies on the matter, the debate on engagement is still very fragmented because the corpus of literature addressing the different areas of engagement is divided and diverse in its nature. In this paper, we discuss the results of a conceptual analysis of the literature conducted in order to investigate overlapping features and areas of divergence among three different areas of investigation and application of the engagement phenomenon in organized settings: the domains of employee engagement, consumer engagement, and patient engagement. These are deliberately selected as prototypical of the phenomenon of engagement along the “inside/outside” of organizational settings. The analysis consisted in a qualitative conceptual survey? Of the scholarly literature indexed with the key terms “employee engagement,” “consumer engagement,” and “patient engagement.” We performed a key-word based survey? Of the literature in the Scopus database. A total of 163 articles were selected and analyzed. The analysis cast light on the following areas of conceptual overlap among employee, consumer and patient engagement: (1) engagement is different from empowerment and activation; (2) engagement is a multi-componential psychological experience; (3) engagement is a self-transformative experience; (4) engagement develops within a relational context; (5) engagement is a systemic phenomenon. These findings, although preliminary and in need of further investigation, suggest the feasibility of promoting a transdisciplinary reflection on the phenomenon of engagement in organized settings.

## Introduction

Organizations are experiencing increased competition, disruptive innovation and continuous changes in their social and economic context (Eldor, 2016). Furthermore, organizations have to deal with a more “critical demand”: clients are more conscious of their rights, more aware of their needs, and more informed about the options to cover such needs. Across sectors and market domains, clients are seeking a more democratic exchange with the organizations that provide services and products for their everyday lives. They are willing to closely examine all the possible options, advantages, and risks implied in different courses of choice, and they demand closer involvement in decision-making related to the coverage of their needs (Cova et al., 2011). Concepts such as *participatory society* (Mossberger et al., 2007; Wallace and Pichler, 2009), *community empowerment* (Perkins and Zimmerman, 1995; Zimmerman, 2000) and *co-creation* (Zimmerman, 2000; Bendapudi and Leone, 2003; Prahalad and Ramaswamy, 2004; Bovaird, 2007) describe the currently evolving sociological and anthropological paradigms driven by people wanting to be greater protagonists of their lives (Hamari et al., 2016; Schor, 2016). Signals which testify to this demand by clients for participation in, and authorship of, the relationship with organizations are retrievable in the different domains of people's daily lives: not only in the area of classic consumption behaviors but also in the healthcare sector, where citizens are more willing to assume an active and participatory role in regard to their health and care management. Today, patients, caregivers, and peers claim their right to judge the adequacy of care received. Moreover, patients rate hospitals and healthcare organizations according to the professionalism of their providers (Graffigna et al., 2016). And the development of new technologies and new forms of communication have allowed this transformation by fostering peer exchanges in many areas of human experience (Kaplan and Haenlein, 2010; Hamari et al., 2016), and by creating new virtual spaces in which it is possible to share information, knowledge and practices related to several domains of human lives.

But this is not all: the decrease of resources (economic and human) in such a demanding context make it imperative for organizations to find new models and strategies so that their service delivery is more sustainable at the economic, environmental and psychological levels. In other words, organizations today are seeking new ways to face the paradoxical need to “do more with less” (Pearson and Clair, 1998; Grewal and Tansuhaj, 2001).

In such a complex scenario, among practitioners as well as in the scholarly literature, the concept of engagement of the individuals involved in organized settings (both as service providers or final receivers) is a promising lever for innovation (Graffigna et al., 2014). In other words, this trend relies on the idea that promoting the active participation, together with the psychological commitment, of people involved in the organizational setting may yield better organizational, relational and psychological sustainability. As a consequence, a growing body of literature, in different disciplinary domains (e.g., management, psychology, sociological sciences, medicine, nursing, political sciences, etc.), has started to address the idea of sustaining people's engagement and participation in organized sectors in order to achieve better organizational sustainability (i.e., employee engagement), improved quality in the relationship with their stakeholders, and general wellbeing and satisfaction among organizations' clients (i.e., consumer engagement and patient engagement in the healthcare domain).

However, despite the number of studies on the matter, the debate on engagement is still very fragmented because the corpus of literature addressing the different domains of engagement is divided and diverse in its nature. It pertains to different disciplines and moves from diverse points of observation. At present, therefore, those bodies of the literature (and thus of organizational practices) do not communicate, with the consequent loss of potential insights and cross-fertilization. Indeed, the literature on employee engagement has rarely crossed the intra-organizational boundaries of its analysis to bridge the level of human resources engagement with the level of clients' engagement with the organization (Kumar and Pansari, 2016). Furthermore, the literature has focused on the engagement of clients receiving services and products from organizations mainly operating in the area of commercial marketing, and it has rarely explored the domain of social and healthcare marketing.

Is engagement therefore an experience that differs according to the organizational domain in which it is experienced and explored? Is engagement a changing phenomenon depending on the role of individuals who have such experience? Or on the contrary are there overlaps among the different areas of investigation and application of the engagement phenomenon (such as employee, consumer and patient engagement)? Is there any ground for starting a transdisciplinary reflection on engagement in organized sectors as a unique and common phenomenon?

In this paper we discuss the results of a conceptual analysis of the literature conducted in order to provide some first answers to the above questions. The idea which inspired this analysis regards the potential of crossing disciplinary boundaries to establish a common, transdisciplinary conceptual foundation for engagement “inside and outside” organizational settings. The goal was ambitious and required conjoint efforts to achieve it. The purpose of this conceptual analysis of the literature was first to explore the feasibility of such interdisciplinary reflection by seeking areas of overlap or divergence among the conceptualizations of employee engagement, consumer engagement, and patient engagement. Other declinations and settings of application of the term “engagement” exist in the literature. We deliberately restricted our investigation to expressions of engagement related to individual actors (i.e., employees, consumers, patients) instead of collective ones (i.e., communities; stakeholders) to explore the phenomenon in its constitutive psychological and relational dimensions. Furthermore, we excluded from our preliminary analysis concepts such as student engagement, technological engagement, and spiritual engagement because they are less related to domain of organizational analysis and to organizations' relations with their adult customers. However, conceptual comparison among all these key terms is valuable and should be further explored in future research.

## Methodology

**Figure 1:**
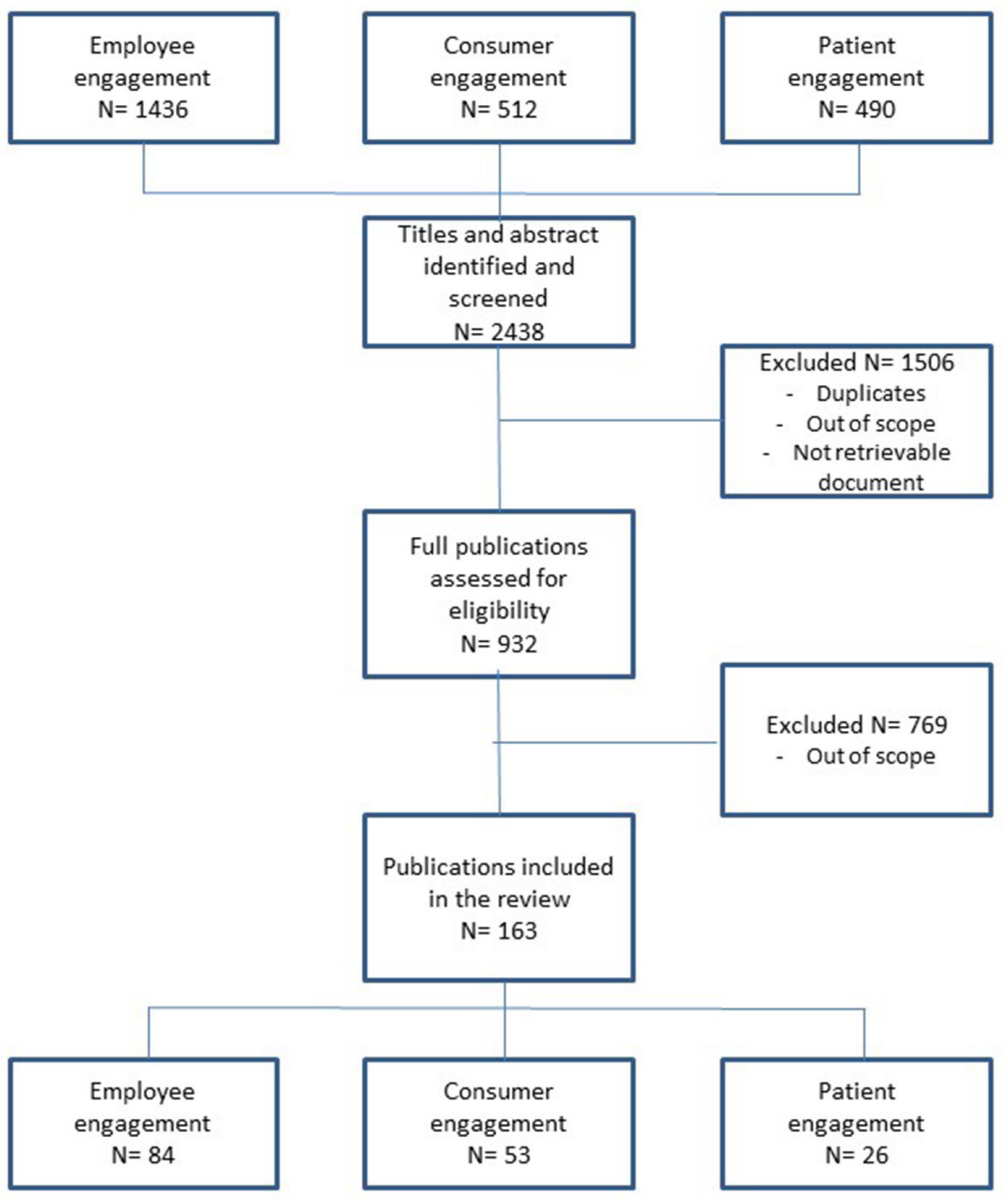
The systematic process of search and analysis of the literature.

This review followed the process of conceptual analysis (Morse, 1995) which followed the process of reference retrieval and analysis described in the next sections (see Figure 1).

### *Search strategy* performed for the conceptual analysis

The survey? Was based on a two-step search process following the advice of Greenhalgh and Peacock (2005): a protocol driven search, and a secondary purposive search of seminal articles.

Initially, a key-word based search was performed in Scopus Database. This database was selected because of its broad and multidisciplinary coverage of scientific literature. We included in the analysis original articles, reviews, and conceptual articles. We opened the analysis to multidisciplinary articles indexed with the terms of interest. Our search was based on the following broad strings of key words: [employe^*^ OR work^*^ OR job^*^ OR consum^*^ OR client^*^ OR costum^*^ OR patient^*^ OR caregiver^*^] AND engagement AND [concept^*^ OR defin^*^ OR theor^*^ OR framework]. We searched for peer-reviewed scholarly papers in the medical, nursing, sociological, psychological, and business literatures. The search was limited to references published in English. We did not impose any time restriction on the search strategy. Due to the large number of articles generated by this search, we limited our analysis to articles presenting the searched-for key words in the title, abstract, and indexed key words.

We then conducted a purposive (“snowball”) search for papers relevant to our analysis based on insights gained from the conceptual analysis performed on the first emerging references. This second phase of the search was performed in other Scientific Databases—Psychinfo, Ebsco, Isi Web of Science, Google Scholar—in order to carry out a broader search of the relevant literature.

### Inclusion criteria

Only manuscripts with a conceptual definition or theoretical framework of employee engagement, patient engagement, and consumer engagement were included in our analysis. We considered as “conceptual” manuscripts which deeply discussed the epistemological underpinnings of the construct/phenomenon under analysis and specified the components of such constructs which should be assessed in empirical research (as suggested by Jaccard and Jacoby, 2010; Castro et al., 2016).

Initially, duplicates were eliminated from the database of references generated by the systematic search. Thereafter, all titles and abstracts were screened to exclude irrelevant records. Finally, the full text of the remaining references was screened to assess if they were eligible for our analysis. We only selected papers which described how the constructs under analysis were understood, described and operationalized. This selection process continued until conceptual saturation was achieved in terms of theoretical understanding and description of the constructs under investigation.

### Analytical process

We based our conceptual analysis on the principles of the Walker and Avant (2005) and Haase et al. (1992) analysis strategies. Following the principles of qualitative content analysis, we performed an in-depth analysis of the retrieved references that included continuous comparison across emerging definitions and continuous validation of the concepts emerging during the process of analysis. This analysis enabled us to clarify the theoretical roots of the concepts under investigation by simultaneously comparing and contrasting the retrieved references in order to understand their inner characteristics, their areas of theoretical overlap, and mutually exclusive attributes. More precisely, the aim of our analysis was to critically appraise the existing definitions of the phenomena under investigation in order to detect their attributes, antecedents, consequences and empirical referents (Castro et al., 2016). The analytical process was recursive and moved through the following steps: (1) The definitions found in the literature were coded and divided into meaningful units. Codes concerned the main areas of investigation: concept definition; attributes, antecedents and consequences. (2) The coding sheet was continuously regenerated to become inclusive of new insights emerging from the analysis. The codes attributed were then revised and clustered into broader categories and themes. (3) The coding sheets produced by the analysis of each construct were then compared and contrasted in order to detect areas of theoretical overlap and divergent features. (4) A final step of interpretation and theoretical abstraction was performed in order to synthesize the main conceptual features of each construct and to build a final comprehensive taxonomy of the overlapping and divergent conceptual areas of the three constructs.

## Results

### Employee engagement

#### Main current definitions of employee engagement

The ongoing debate on employee engagement (and related synonyms such as “work engagement” and “job engagement”) began in the early 1990s. Although there exist many empirical investigations and theoretical studies on these concepts, the scholarly debate still presents some areas of opacity and theoretical gaps. Several definitions of employee engagement exist, and no single shared framework has been established. Furthermore, scholars point out the separation and poor dialogue between the scholarly literature and the managerial debate on this concept, resulting in multiple and divergent definitions of employee engagement, together with poorly shared and structured strategies to promote it. Furthermore, there is still controversy on the conceptual boundaries between employee engagement and other related organizational constructs, such as psychological empowerment, organizational commitment, job satisfaction; job involvement and job affect (Dalal et al., 2008; Newman and Harrison, 2008; Shuck, 2011; Shuck et al., 2013; Eldor, 2016). Some critical scholars have even argued that employee engagement is nothing more than a “*new blend of old wines*” (Newman et al., 2010). In this context of open debate, in order to lay the bases for a conceptual analysis of employee engagement, in the following sections we review the most established and cited definitions of the concept (see Table 1). Schaufeli et al. (2002) define the concept as “*a positive, fulfilling work related state of mind that is characterized by vigor, dedication, and absorption*” and they oppose this state of mind with the contrary experience of burnout, in line with Maslach and Leiter's (1997) view of engagement as “a persistent positive affective state characterized by high levels of activation *and pleasure*,” whereas the experience of burnout is characterized by parallel but opposite dimensions such as exhaustion, cynicism and ineffectiveness. Macey and Schneider (2008) define engagement at work as “*a desirable condition*, [which] *has an organizational purpose, and connotes involvement, commitment, passion, enthusiasm, focused effort and energy, so it has both attitudinal, and behavioral component*s”: the experience of involvement, passion, enthusiasm and energy lived by the employee. The managerial literature mainly tends to associate employee engagement with work satisfaction, still using the Gallup conceptualization of work engagement. For instance, Harter et al. (2002) define the concept as the “*individual's involvement and satisfaction with, as well as enthusiasm for, work*,” while Rothbard (2001) defines work engagement as “*one's psychological presence in or focus on role* activities.” Still highly influential in the debate on employee engagement is the seminal conceptualization of Kahn (1990), who first defined work engagement as “*the harnessing of organizations members' selves to their work roles, by which they employ and express themselves physically, cognitively, and emotionally during role performance*.” Building on this seminal definition, Shuck and Wollard (2010) broadened the conceptualization of work engagement by stating that it reflects the holistic and simultaneous expression of individuals' physical, emotional and cognitive energy in their work roles. Particularly, they define work engagement as “*an individual employee's cognitive, emotional and behavioral state directed toward desired organizational outcomes*.” Rich et al. (2010) extended Khan's definition further by stating that there are three subcomponents of work engagement: physical engagement, emotional engagement, and cognitive engagement. More recently Eldor (2016) has defined work engagement as “*a combination of individuals' deeply physical, emotional and cognitive connectedness with the significant facets of their lives: work, personal life, and community*” which is crucial for providing an organization with a competitive advantage.

**Table 1:** Definitions of “employee engagement.”

**References**	
Schaufeli et al., 2002, p. 74	“a positive, fulfilling work-related state of mind that is characterized by “vigor, dedication and absorption”; this experience is opposed to the contrary experience of burnout”
Maslach and Leiter's, 1997, p. 417	as “a persistent positive affective state characterized by high levels of activation and pleasure,” whereas the experience of burnout is characterized by parallel but opposite dimensions such as exhaustion, cynicism and ineffectiveness
Macey and Schneider, 2008	“a desirable condition, [which] has an organizational purpose, and connotes involvement, commitment, passion, enthusiasm, focused effort and energy, so it has both attitudinal and behavioral components”: the experience of involvement, passion enthusiasm and energy lived by the employee
Harter et al., 2002	individual's involvement and satisfaction with, as well as enthusiasm for, work
Rothbard, 2001, p. 656	“one's psychological presence in or focus on role activities”
Kahn, 1990, p. 694	“the harnessing of organizations members' selves to their work roles, by which they employ and express themselves physically, cognitively and emotionally during role performance”
Shuck and Wollard, 2010, p. 103	“an individual employee's cognitive, emotional and behavioral state directed toward desired organizational outcomes”
Rich et al., 2010	there are three subcomponents of work engagement: physical engagement, emotional engagement and cognitive engagement
Eldor, 2016, p. 332	“a combination of individuals' deeply physical, emotional and cognitive connectedness with the significant facets of their lives: work, personal life and community”

#### Attributes of employee engagement

Some constitutive attributes of employee engagement seem to recur in the literature analyzed.

##### Multidimensionality

Authors generally agree in defining employee's engagement as a complex and multidimensional psychological experience of the individual at work. Particularly, engagement is articulated into three main psychological components: cognitive engagement, affective engagement, and behavioral engagement. These three dimensions appear to overarch all the specific psychological manifestations of the engagement experience. Furthermore, in order to make the experience of engagement effective, the three dimensions should be consistent and simultaneously implied.

##### Relationality

The scholarly debate agrees in positioning employee engagement within the broader debate on organizational relationships, together with kindred concepts (such as psychological empowerment, organizational commitment Mowday et al., 1979, and job satisfaction Locke, 1969). The concept of employee engagement, indeed, is deeply relational in its nature. Although it denotes an individual trait or state (Macey and Schneider, 2008), it is always described as the result of the type of contact and exchange that an individual may entertain, both at the psychological and performance levels, with his/her job task and the overarching organizational setting in which this is performed.

##### Individual identity

In the seminal theorization of Kahn (1990) subsequently resumed by other scholars, employee engagement is linked to individuals' self-expression and self-actualization. In particular, engagement is a function of how an individual lives, expresses and attributes meaning to his/her job role. Furthermore, the expression of employee role identity in the definition of engagement does not seem to be detached from the other roles experienced by the individual. Definitions of employee engagement suggest that good integration among different self domains into a more coherent experience of self-identity is crucial for work engagement. Work engagement is often described as the consequence of individual personal development.

##### Driving

In all theorizations, work engagement is described as a “force” driving employees' performance. The motivational nature of engagement emerges from the literature analyzed, and it is also retrievable in the empirical studies which assess the role of engagement in predicting the quality and quantity of job performances and organizational competitiveness. Furthermore, empirical investigations often treat work engagement as a mediator or a moderator of other variables in employees' or organizations' performances.

#### Antecedents of employee engagement

Several empirical studies have attempted to explore and model the relationship between engagement and several types of predictor. Our analysis made it possible to cluster the main recurrent precursors of employee engagement into the following three categories.

##### Individual resources

There is much debate on the individual characteristics which may sustain or hinder work engagement (Bakker and Demerouti, 2008; Bakker and Leiter, 2010). For instance, individuals' attitudes toward their jobs (Mackay et al., 2017); individuals' perceptions of closeness and alignment with organizational values and mission (Ilkhanizadeh and Karatepe, 2017); employees' level of self-efficacy and perceived control over their jobs (Noblet et al., 2016; Chmiel et al., 2017) employees' goal orientation (Adriaenssens et al., 2015), optimism, personal capital (Karatepe and Karadas, 2015), and psychological empowerment (Kimura, 2011); and employees' dedication to task completion (Porter, 1996; Eldor and Harpaz, 2016) are also considered precursors of employees' engagement.

##### Job resources

Studies have demonstrated a positive relationship between various features of the job and employees' engagement. These features are, for instance, the learning climate, the level of autonomy, role fit, job control, task significance, the supervisor's support and feedback, and task variety (Schaufeli and Bakker, 2004; Hakanen et al., 2006; Bakker and Demerouti, 2007; Crawford et al., 2010; Halbesleben, 2010; Shuck, 2011; Eldor and Harpaz, 2016).

##### Organizational resources

Other potential antecedents of work engagement are attributable to organization characteristics, such as organizational climate, learning climate, organizational structure, and quality of the interpersonal relationships among colleagues and between different roles (Schaufeli et al., 2009; Kimura, 2011).

#### Consequences of employee engagement

The broad interest of scholarly as well as practitioners' debate in employee empowerment is largely due to the intra-organizational and extra-organizational outcomes that it is supposed to promote. Although final agreement on the main outcomes of employee engagement has still to be reached, and some scholars even point to the lack of knowledge about the added value of employee engagement in promoting organizations' competitive advantage (Bakker et al., 2011; Eldor, 2016), some general domains of employee engagement appear recurrent in the literature analyzed.

##### Employee performance

Studies have demonstrated that a high level of work engagement predicts a greater commitment of employees to their job tasks, by also improved effectiveness in task completion (Rich et al., 2010; Shuck, 2011, and the perceived satisfaction with one's own work. Furthermore, highly engaged employees are likely to solve or overcome organizational obstacles (Ulrich, 1994; Barney and Wright, 1998; Gorton and Schmid, 2004).

##### Organizational performance

The engagement of employees has also been discussed as a potential predictor of organizational outcomes, such as level of customer satisfaction (Salanova et al., 2005), sales improvement (Xanthopoulou et al., 2008; Demerouti and Cropanzano, 2010), innovation (Hakanen et al., 2008), and costs reduction (Eldor, 2016).

##### Personal fulfillment

Furthermore, work engagement is linked to extra-organizational outcomes primarily to do with individuals' self-actualization and well-being. A seminal study by Schaufeli and colleagues demonstrated the linkage between work engagement and individuals' positive emotions and well-being perception (Menezes De Lucena Carvalho et al., 2006; Bakker and Demerouti, 2008; Macey and Schneider, 2008; Shimazu et al., 2016). Furthermore, studies have highlighted the influence of work engagement on employees' perception of quality of life, their satisfaction with life, and generally their overall ability to integrate in the community of reference and assume a satisfactory social role.

### Consumer engagement

#### Main current definitions of consumer engagement

Consumer engagement is today considered an important milestone for post-modern marketing, as testified by the topic's growing coverage by the managerial as well as scholarly literature (Gambetti and Graffigna, 2010). However, the theoretical foundation for this concept is at its beginnings, and a shared and solid background is still lacking (France et al., 2016; Harmeling et al., 2017). Also in the case of consumer engagement, some scholars have pointed to the risk that it is an “old wine in a new bottle” (Brodie et al., 2011; Harmeling et al., 2015), thus using the same critical metaphor as applied in the employee engagement literature. The opacity in definition of the concept is also related to the multiple marketing settings in which engagement is considered and discussed: the vast majority of studies have assessed the phenomenon in the domain of consumer-brand relationships (Bowden, 2009; Graffigna and Gambetti, 2015). Others, particularly managerial studies, have mainly focused on engagement toward media communication and advertising (Calder and Malthouse, 2005; Wang, 2006; Kilger and Romer, 2007; Heath, 2009; Hennig-Thurau et al., 2010; Tafesse and Tafesse, 2016). Finally, Harmeling et al. (2015) have suggested that a distinction should be drawn between consumer engagement (as an outcome of marketing actions) and engagement marketing (as all the specific marketing strategies enacted by a company to produce consumer engagement).

The majority of studies (see Table 2) describe consumer engagement as a multidimensional phenomenon which encompasses cognitive, affective and behavioral components of consumers' experience (Bowden, 2009; Brodie et al., 2011; Hollebeek, 2011). Hollebeek and Chen (2014) defined consumer engagement as the level of a consumer's cognitive, emotional and behavioral investment in specific brand interactions (Hollebeek, 2011; Hollebeek and Chen, 2014; Hollebeek et al., 2016). However, although scholars tend to agree on the complex and multifaceted nature of consumer engagement, contributions in the literature tend to focus on isolated and specific dimensions of it. For instance, several authors have based the definition of consumer engagement on its behavioral manifestations, which are seen as more objective and explorable (Goldsmith et al., 2010, 2011; Van Doorn et al., 2010; Verhoef et al., 2010; Jaakkola and Alexander, 2014; Verleye et al., 2014). Others argue for considering the psychological complexity that lies behind consumer engagement. They define it as a psychological state of mind and discuss its cognitive and emotional components (Heath, 2009; France et al., 2016). However, the nature of consumer engagement is still a matter of debate. Some scholars argue that it should be considered a state of mind circumscribed to a specific moment and setting of contact with the brand/company (Sprott et al., 2009). Others suggest a broader definition as a complex process which develops in time along the consumer experience journey (Bowden, 2009; Brodie et al., 2011; Gambetti et al., 2012; Graffigna and Gambetti, 2015; Maslowska et al., 2016). Finally, similarly to the case of scholarly debate on employee engagement, the conceptual boundaries between consumer engagement and other related concepts such as consumer empowerment (Rapp et al., 1993; Tiu Wright et al., 2006), consumer involvement (Gilles and Kapferer, 1985; Mittal, 1995), consumer commitment (Moorman et al., 1992; Morgan and Hunt, 1994), brand attachment (Thomson et al., 2005; Smaoui, 2008; Whan Park et al., 2010) and brand experience, still remain matters to be debated.

**Table 2:** Definitions of “consumer engagement.”

**References**	
Kumar and Pansari, 2016, p. 2	“the attitude, behavior, the level of connectedness (1) among customers, (2) between customers and employees, and (3) of customers and employees within a firm”
Hollebeek et al., 2016, p. 6	A customer's motivationally driven, volitional investment of focal operant, resources (including cognitive, emotional, behavioral, and social knowledge and skills), and operand resources (e.g., equipment) into brand interactions in service systems”
Jaakkola and Alexander, 2014	A customer's motivationally driven, volitional investment of focal operant resources (including cognitive, emotional, behavioral, and social knowledge and skills), and operand resources (e.g., equipment) into brand interactions in service systems (p. 6)
Verleye et al., 2014, p. 69	“Voluntary, discretionary customer behaviors with a firm focus…customers' interactive, cocreative experiences with a firm”
Vivek et al., 2012, p. 127	“Beyond the purchase…events and activities engaged in by the consumer that are not directly related to search, alternative evaluation and decision making involving brand choice”
Brodie et al., 2011, p. 9	“Psychological state that occurs by virtue of interactive, cocreative customer experiences with a focal agent/object (e.g., a brand) in focal service relationships”
Hollebeek, 2011, p. 790	The level of an individual customer's motivational, brand-related and context-dependent state of mind characterized by specific levels of cognitive, emotional and behavioral activity in direct brand interactions”
Bijmolt et al., 2010, p. 341	“Customers can cocreate? value, cocreate competitive strategy, collaborate in the firm's innovation process, and become endogenous to the firm”
Kumar et al., 2010, p. 297	“Customers contribute to firms in many ways that are beyond direct transactions”
Van Doorn et al., 2010, p. 253	“Customer behavioral manifestations toward the brand or firm, beyond purchase”
Verhoef et al., 2010, p. 247	“A behavioral manifestation toward the brand or firm that goes beyond transactions”
Gambetti et al., 2012	“CBE as characterized by three relational phases marked by increasing levels of brand enacting that are related to the brand ability to progressively “approach” its consumers, building with them a bond which shows a growing relationship strength. In the first phase of the CBE process the brand reveals its appearance to consumers, then in the second its body, and finally in the third its soul”
Sprott et al., 2009, p. 92	“BESC is the consumers' tendency to include important brands as part of their self-concept”
Bowden, 2009, pp. 64–66	The process of engagement traces the temporal development of loyalty by mapping the relationships between the constructs of calculative commitment, affective commitment, involvement, and trust as customers progress from being new to a brand to becoming repeat purchasers of a specific brand
Calder and Malthouse, 2005	“Media engagement takes into account the effectiveness of a message and the environment within which that message is presented”
Wang, 2006, pp. 356–358	“Engagement is defined as a measure of the contextual relevance in which a brand's messages are framed and presented based on its surrounding context”
Graffigna and Gambetti, 2015	“CBE seems to be shaped by a complex psychological contact experience between consumer and brand, in which the brand gets incorporated in consumers' imagery, social networking and inter-generational life experiences by acting as their “dream carrier,” “relationship facilitator” and “compass”

#### Attributes of consumer engagement

##### Psychological ownership and self-transformation

The phenomenon of engagement is described as a “self-transformation” (Harmeling et al., 2015) of the consumer, in the sense that s/he deliberately becomes an active agent in the relationship with the brand (Van Doorn et al., 2010) but also that s/he incorporates attributes of the brand itself into personal self-expression and determination (Sprott et al., 2009). Graffigna and Gambetti (2015) also postulated that consumer engagement coincides with consumers' self-transformation and achievement of a better balance in the brand relationship in a renewed “eudaimonic project.”

##### Multicomponentiality

Most studies view consumer brand engagement as a consumer's multidimensional activation state with cognitive, affective and behavioral components (Bowden, 2009; Brodie et al., 2011; Hollebeek, 2011) and influenced by motivational drivers (Calder and Malthouse, 2005; Van Doorn et al., 2010; Malthouse and Peck, 2011). In this regard, consumer brand engagement has been defined as the level of a consumer's cognitive, emotional and behavioral investment in specific brand interactions (Hollebeek and Chen, 2014).

##### Intentionality

The motivational nature of engagement is a recurrent topic in the scholarly debate: consumer engagement is described as a propulsive and positive energy that links consumers and brand in pursuit of? Shared goals of brand/product performance and experience. In particular, consumer engagement is often described as the consequence of a deliberative positive attitude (and conduct) toward the company (Calder and Malthouse, 2005; Malthouse and Peck, 2011). In a seminal manuscript, for instance, Van Doorn et al. (2010) defined consumer engagement as the “consumer's behavioral manifestation toward a brand or firm, beyond purchase, resulting from motivational drivers.”

##### Relationality

Bowden (2009) was the first to posit analysis of consumer engagement within of the consumer/brand relationship. Also more recent studies read consumer engagement in light? Of individuals' interaction with a company: for instance, Kumar and Pansari (2016) describe consumer engagement in terms of three levels of connectedness: (1) among customers, (2) between customers and employees, and (3) of customers and employees within a firm. Furthermore, Verleye et al. (2014) describe consumer engagement as “*voluntary, discretionary customer behaviors with firm focus…customers' interactive, co-creative experience with a firm*.”

#### Antecedents of consumer engagement

##### Consumer resources

The level of consumer psychological empowerment and self-confidence is considered to be a precursor of engagement behavior (Füller et al., 2009; Tsai and Men, 2013; Morrongiello et al., 2017). Moreover, generally the level of consumer motivation, is considered crucial in generating engagement (Van Doorn et al., 2010). Recently, Harmeling et al. (2015) have also argued for the importance of other consumer resources for engagement, such consumers' networks (defined in terms of consumers' interpersonal ties); *consumer persuasion capital* (defined in terms of “degree of trust, goodwill and influence” of a customer on other potential costumers); *customer knowledge stores* (in term of accumulation of specific knowledge related to the company and the brand); and *customer creativity* (in terms of capacity to contribute with novel ideas to marketing decision making).

##### Brand features

How brand features and “brand personality” impact on consumer loyalty and consumer brand attachment is much debated in the scholarly as well as managerial literature (Chaudhuri and Holbrook, 2002; Malär et al., 2011). Consistently, the ability of the brand to generate emotional attachment is considered to be one of the antecedents of consumer engagement (Morrongiello et al., 2017). Furthermore, the experiential dimension of the consumer-brand touch point and the ability of the brand to be perceived as close to consumers' values and preferences are other elements considered crucial for the development of engagement (Gambetti et al., 2012; Graffigna and Gambetti, 2015).

##### Marketing initiatives

Consumer engagement has also been widely discussed as the consequence of deliberate marketing efforts, such as purchase incentives, co-creative platforms, and better interactive communication between consumers and brands (Fuchs et al., 2010; Kozinets et al., 2010; Gambetti et al., 2016). Harmeling et al. (2015) argue for an “engagement marketing” as a specific set of marketing initiatives aimed at increasing consumer engagement, considered as one of the outcomes of “engagement marketing.”

#### Consequences of consumer engagement

##### Company revenue

Some studies demonstrate that high levels of consumer engagement are linked to increased revenue and lower costs (Fuchs and Schreier, 2011; Schmitt et al., 2011) and increased decision making and purchase behaviors (Ramani and Kumar, 2008; Bowden, 2009; Sprott et al., 2009; Van Doorn et al., 2010; Kumar and Pansari, 2016).

##### Consumers' loyalty

Studies claim that consumer engagement is related to an enhanced brand equity (Hoeffler and Keller, 2002; Schultz and Block, 2011) and generally to high consumer retention. Some studies have also highlighted that high levels of consumer engagement are related to better satisfaction and affection in the customer?-Brand relationship, enhanced brand trust, and consumer commitment to the company (De Matos and Rossi, 2008; Bowden, 2009; Gambetti and Graffigna, 2010; Hollebeek, 2011).

##### Word of mouth and advocacy

Furthermore, consumer engagement has also been associated with an increased tendency of consumers to advocate a brand in their peer-networks, mainly through social media (Kozinets et al., 2010; Libai et al., 2010; Brodie et al., 2013). Scholars have also focused on consumer-to-consumer interactions and consumer-brand interactions in online communities as functions of engagement and as sources of value co-creation (Brodie et al., 2013), and the generation of new ideas and solutions which may contribute to new product development and better marketing strategies (Van Doorn et al., 2010; Harmeling et al., 2017; Morrongiello et al., 2017).

### Patient engagement

#### Main current definitions of patient engagement

The literature debate on patient engagement started later than those on employee and consumer engagement, and its beginning dates to only about a decade ago (Barello et al., 2012). However, interest in the concept, again in both the scholarly and the practitioners' literature, has greatly increased in recent years (see Table 3). Despite this growing production of studies on the topic, however, no consensus has yet been reached on a shared definition of patient engagement, and several perspectives coexist. Furthermore, it is interesting that in the case of patient engagement (more than in the case of the concepts previously analyzed) several different disciplines have approached the topic (i.e., medicine, nursing, political science, management, psychology, social science, and even computer science!, see e.g., Barello et al., 2014). This unavoidably leads to a richness of perspectives, but also to the risk of a “Babel” of different languages, epistemological underpinnings, and sensibilities. In particular, the different definitions of patient engagement available in the literature appear to focus on different levels of the engagement phenomenon (Fumagalli et al., 2015). In other words, the majority of authors consider engagement in terms of a behavioral state of the individual (e.g., Gruman et al., 2010), mainly overlapping it with patients' skills in self-managing their health and care. A few other scholars have attempted to provide a theoretical foundation for the dimensions involved in the experience of “engagement” and the developmental process of such subjective experience (i.e., Hibbard et al., 2009; Graffigna et al., 2014). Finally, others have proposed a more systemic definition of patient engagement in an attempt to describe all its organizational components (Carman et al., 2013; McCormack et al., 2017).

**Table 3:** Definitions of “patient engagement.”

**References**	
Hibbard et al., 2009	Patients' motivation, knowledge, skills, and confidence to make effective decisions to manage their health.
Gruman et al., 2010	Set of behaviors including two overarching domains: (1) “managing health” behaviors, which is both the self-management of chronic disease and the adoption of healthy behaviors, and (2) “managing healthcare” behaviors, which can be both patient and “consumeristic” behaviors.
Graffigna et al., 2014	Process-like and multi-dimensional experience, resulting from the conjoint cognitive (think), emotional (feel) and conative (act) enactment of individuals toward their health management. In this process patients go through four successive phases (disengagement, arousal, adhesion and eudaimonic project). The unachieved synergy among the different subjective dimensions (think, feel, act) at each stage of the process may inhibit patients' ability to engage in their care.
Légaré and Witteman, 2013	[“engagement” is] the process of individuals' responsabilization that ensures that clear information lead to the best decision for the person who is seeking the care thus improving self-management.
Mittler et al., 2013	Engaging consumers refers to the performance of specific behaviors (“engaged behaviors”) and/or an individual's capacity and motivation to perform these behaviors (“activation”) aimed at gaining health.
Mahmud, 2004	It is a process that leads to setting healthcare priorities. It consists in empowering people to provide input in decisions that affect their lives and encourages support for those decisions, which in turn improves the public's trust and confidence in the healthcare system.
Dearing et al., 2005	Developing “engagement” means fostering those client-therapist working alliances that help the client gain a more realistic understanding of the nature, process, and expected outcomes of treatment.
Davis et al., 2007	Option for patients to be informed partners in their care, including a recasting of the care relationship where clinicians enact the role of adviser, and patients or designated surrogates for incapacitated patients serving as the locus of decision making.
McBride and Korczak, 2007	It is a process that will allow, at different levels, the wider community to have a say in the future direction of their health.
Dunston et al., 2009	Dialogic and co-productive partnership between health system, health professionals and citizen/health consumers through which these actors become co-productive.
Forbat et al., 2009	[engaging patients means] working in partnership with service-users having them inform (i) service redesign/improvement, (ii) policy, (iii) research, and (iv) their own care/treatment. It also implies balancing powers among patients and health providers.
Schley et al., 2012	Engaging clients in the therapeutic encounter means developing collaboration, perceived usefulness, and client-therapist positive interaction.
Mulley et al., 2012	Process of shared decision making described as a sequence of three types of conversation: team talk, option talk and decision talk. [Engaging patients] means creating a preference diagnosis which has a unique profile of risks, benefits and side effects.
Sanders and Kirby, 2012	A collaborative, bidirectional process whereby patients' knowledge and experience is shared in a dialogue with program developers, health practitioners and researchers. It involves actively harnessing the consumer's voice to strengthen the quality, relevance and effectiveness of an intervention.
Carman et al., 2013	Shared power and responsibility among the actors of the care process where (i) the patient becomes an active partner in defining agendas and making decisions; (ii) the information flow is bidirectional; (iii) patients act also as representatives of consumer organizations.
Patel and Rajasingam, 2013	The [engaged] patients have the ability to balance clinical information and professional advice with their own needs and preferences. It is a collaborative approach where shared decision making, equal distribution of power and exchange of clinical information are enacted.
Higgins et al., 2017	“individual desires and capabilities, partnering with providers and institution maintaining the power hierarchy and increasing the confidence and skill levels of patients.”

Hibbard et al. (2009) describe “engagement” as “the patients' motivation, knowledge, skills, and confidence to make effective decisions to manage their health.” This framework starts from the consideration that the level of “engagement” may affect individuals' healthcare choices and daily disease management, thus having an impact on healthcare utilization, costs and clinical outcomes. Four gradients of “engagement” have been identified by these authors, although they are not described at the psycho-social and clinical level. Furthermore, also in this case, the conceptualization fails in explaining of the dynamicity that governs the increase in patients' “engagement.” Gruman et al. (2010) for instance, describe “engagement” as a set of behaviors and actions that allow individuals to effectively manage their health and healthcare in order to obtain the greatest benefits. This definition emphasizes the role of individuals in shaping their behaviors in order to interact in the best way possible with the health care. But it appears merely taxonomic and does not offer insights into the dynamics occurring when individuals engage in their health care. Moreover, this proposal reduces the concept of “engagement” to its behavioral manifestations alone. Carman et al. (2013) provide a more dynamic conceptualization of “engagement” as a continuum of possible interactions between the patients and the healthcare system (i.e., from “consultation” to “partnership” to “shared leadership”). This definition of patient “engagement” has the indubitable strength of considering “engagement” as a systemic phenomenon which is the outcome of actions carried out at different levels of complexity (i.e., individual, relational, communitarian, organizational, and health policy). However, also this conceptualization is insufficient because it reduces the “engagement” process to merely the behavioral/conative dimensions of the patient's experience, and because it does not explain the dimensions that may sustain or inhibit the passage from one stage to the other of the continuum. Graffigna et al. (2014) define “engagement” as a “process-like and multi-dimensional experience, resulting from the conjoint cognitive (think), emotional (feel) and conative (act) enactment of individuals toward their health management.” In this process, patients go through four successive phases (i.e., “disengagement,” “arousal,” “adhesion,” and “eudaimonic project”). In their conceptual framework, these authors discuss “engagement” as a process, and they describe its different phases in psychological terms. However, also this model appears weak in explaining the dynamic passage from one to the other phase of the process. Furthermore, it fails to define? The contextual and organizational elements that may sustain or inhibit the “engagement” process. Finally, Higgins et al. (2017) propose considering engagement as an encompassing level of patients' participation in health care, since engagement relies on “individual desires and capabilities, partnering with providers and institution maintaining the power hierarchy and increasing the confidence and skill levels of patients.” In particular, the authors consider the attributes of care personalization, access, commitment, and therapeutic alliance as constitutive of the engagement phenomenon.

#### Attributes of patient engagement

##### Self-determination

Patient engagement is described as the result of the individual's choice to change his/her attitudes and behaviors toward health care. Furthermore, patient engagement has also been discussed as an ethical principle for modern medicine, in order to give voice to patients regarding their needs and preferences in the care journey, and thus to enhance their self-determination rights also in health care. Hibbard (2017) has defined the state of engagement and activation as the patient's self-perception of his/her self as agent in the healthcare process. Graffigna et al. (in press) further elaborated on this point by defining patient engagement as the change of the patient's “role identity” along the healthcare journey: from passive recipient to co-author of the care process.

##### Complexity

As in the case of the previously analyzed concepts, also patient engagement is described as a complex phenomenon which involves several dimensions of individuals' functioning, as well as different actors. Authors who focus on the individual's experience of engagement, describe this as the result of several psychological dimensions. Shared by the various definitions is the assertion that engagement results from a conjoint and parallel activation of the individual at the cognitive, behavioral and emotional levels. The large number of studies on the intra-individual and psychological factors involved in the engagement process confirm this variety of components. Other authors assume a broader perspective in defining engagement by detecting several organizational components and layers of analysis which determine it. These authors propose to consider patient engagement as the result of several factors belonging to healthcare organizational functioning and the surrounding socio and economical context. *Driving nature:* patient engagement, too, is considered and studied as the potential driver of change in patients' clinical behavior and in healthcare organizational performances. At the individual level, scholars tend to link the experience of engagement to a sort of motivational lever which drives patients to change their attitudes and conduct in healthcare. Also authors who assume a more systemic and organizational perspective agree that engagement is a potential source of change and enactment of improved organizational processes.

#### Antecedents of patient engagement

##### Relational factors

Some authors describe engagement as a function of the dyadic patient/physician relationship. They thus restrict the concept of “engagement” to the domain of therapeutic alliance (Higgins et al., 2017) and shared decision making (i.e., Davis et al., 2007; Mulley et al., 2012). Other studies suggest a broader vision of inter-individual factors at the basis of “engagement”: in particular, they focus on the role of complex networks of peer-to-peer exchanges (i.e., Dunston et al., 2009) and on the dialogue between the citizen and the healthcare system conceived as a whole (Mahmud, 2004; McBride and Korczak, 2007).

##### Intra-individual factors

Studies have highlighted the intra-individual factors involved in shaping “engagement,” more often with exclusive reference to the cognitive aspects of self-efficacy, perceived locus of control, and health literacy (Légaré and Witteman, 2013; Mittler et al., 2013; Smith et al., 2013; Prey et al., 2014). Furthermore, some authors propose engagement as the consequence of patients' level of empowerment (Fumagalli et al., 2015). Some authors have also highlighted the role of positive emotions and psychological elaboration of the illness condition as predictors of patient engagement (McCusker et al., 2016; Prey et al., 2016). By contrast, there is less agreement in the literature on the association between patient engagement and socio-cultural characteristics of the individual such as gender, age, level of education (Hibbard et al., 2008; Bos-Touwen et al., 2015) and level of income (Skolasky et al., 2008; Rask et al., 2009).

##### Organizational factors

Personalization of the care approach (Higgins et al., 2017) and the level of patient centricity assumed by the healthcare organization (Borghi et al., 2016) are discussed as crucial antecedents of patient engagement. Furthermore, the issue of access to the healthcare system and to the needed resources is considered a fundamental factor influencing engagement, as well as the level of perceived quality of care (Carman et al., 2013).

#### Consequences of patient engagement

##### Improved patients' health management and better clinical outcomes

There are indications that patient engagement in the care process may indeed improve health outcomes across disease conditions (Saft et al., 2008; Munson et al., 2009; Green et al., 2010; Stepleman et al., 2010; Begum et al., 2011; Skolasky et al., 2011; Alexander et al., 2012). For example, studies have found that patients who were actively involved in their care plans were more likely to trust their clinicians (Becker and Roblin, 2008), more likely to adhere to treatment prescriptions (Hibbard et al., 2008) and less likely to experience adverse clinical events and hospital readmissions (Hibbard et al., 2008). Patient engagement also seems to contribute to fostering sustainable lifestyles and avoiding unsafe conduct (Jordan et al., 2008; Hibbard, 2009; Reid et al., 2010; Cosgrove et al., 2013).

##### Improved patients' satisfaction and quality of life

Patient engagement is helpful in fostering personal growth and integration not only into the healthcare environment but also into the reference community by promoting satisfaction, opportunities for action, and self-expression (Martinez et al., 2009; Heesen et al., 2011; Bolderston, 2016). Furthermore, it may contribute to enhancing quality of life with the goal of increasing wellness and generating strengths and resilience in individuals after acute events (Haywood et al., 2017).

##### Healthcare costs reduction

The patient's engagement, in terms of better sensitization, knowledge, and empowerment in his/her process of care and cure, thus seems to be crucial for achieving an efficient balance between the increase in healthcare demand and the reduction of economic resources allocated to the healthcare system in all mature societies today (Laurance et al., 2014; Graffigna et al., 2016). Furthermore, patient engagement may not only contribute to reducing the direct costs of the healthcare system; it may also concur in (re)orienting economic resources in the management of healthcare systems to reduce waste of money (Fisher et al., 2011; Hibbard and Greene, 2013).

## Discussion

### Employee, consumer, and patient engagement: divergences and overlaps

The foregoing conceptual analysis, although preliminary and partial, has cast light on some interesting conceptual areas of overlap as well as divergence among the phenomenon of employee engagement, consumer engagement, and patient engagement selected as expressions of different positions and experiences of engagement along the “insid/outside” organizational continuum. Our research was prompted by an interest in exploring the feasibility and proposability of transdisciplinary reflection on engagement in organized settings.

Although flourishing, the literature debate on these concepts appears fragmented and distant from a shared and clear theoretical conceptualization. In all cases, the pragmatic importance of the engagement concept seems to drive the ongoing reflection, sometimes leading to a lack of academic consensus. To contribute to grounding a common sensibility on these topics we propose here some interpretative keys which appear recurrent among the three concepts.

### First proposition: engagement is different from empowerment and activation

In general terms, engagement may be described as a specific psychological experience which differs from those of psychological empowerment and activation due to its relational nature and its broad spectrum of influence at the micro, meso and macro levels of an organizational system.

Although across the three corpuses of literature analyzed, the potential intertwining between engagement and other psychological concepts such as empowerment and activation is still a matter of debate, the majority of scholars seem to consider such concepts as diverse and autonomous, whereas they are interrelated. In the literature on employee and consumer engagement, empowerment is proposed as an antecedent of engagement, whereas activation is considered to be a potential behavioral outcome of it (Eldor, 2016; Harmeling et al., 2017). In the literature on patient engagement, empowerment and activation are often treated as overlapping phenomena, and engagement is generally viewed as an encompassing concept, or rather as a predictor empowerment or activation (Menichetti et al., 2014; Fumagalli et al., 2015).

The areas of contradiction and debate still present in the literature suggest that the analysis of the conceptual boundaries among engagement, empowerment and activation should be envisaged as a future priority line of investigation in this field. However, this also testifies to the tendency, both in managerial practice and empirical research, to consider engagement as a new and original concept which needs specific foundation and assessment.

### Second proposition: engagement is a multi-componential psychological experience

A large amount of empirical research has been conducted over the past three decades to assess the inner components of employee, consumer and patient engagement, their antecedents and their consequences. Although not only the psychological literature has conducted analysis of engagement in these different settings, all authors seem to assume that engagement relates to a specific individual psychological experience. However, there is no agreement among scholars in regard to the nature of such experience: in the literature analyzed, some authors describe it as a state of mind, others as an individual trait (Macey and Schneider, 2008). In regard to patient engagement and consumer engagement, some authors propose viewing engagement as a psychological process articulated into incremental levels or positions (Hibbard, 2009; Carman et al., 2013; Graffigna et al., 2014). However, further debate on this point is needed across areas of engagement investigation.

Nevertheless, it appears established (particularly in the literature on employee engagement and patient engagement) that engagement should be viewed as a multi-componential psychological experience which affects the individual at several levels of his/her functioning: emotional, cognitive and behavioral. Furthermore, seminal studies in the domain of employee and patient engagement have also pointed out that engagement results from a good balance and conjoint activation of all these three dimensions of the individual's functioning.

Furthermore, no consensual developmental model of engagement seems to exist: the established theories and their related assessment measures seem to more concerned with determining the various factors describing engagement than with describing where the individual is positioned in his/her engagement experience. In other words, there is still scant consideration of the potential dynamic process leading an individual to acquire a growing degree of engagement along his/her course of experience. In particular, in the literature on employee engagement, no dynamic conceptualization of engagement was retrieved, apart from Schaufeli et al.'s (2002) conceptualization of engagement as the opposite pole on a continuum with burnout. Similarly, consumer engagement is mainly described by authors as a state of mind or a behavioral response to marketing actions (Van Doorn et al., 2010; Harmeling et al., 2017). But some studies have discussed the importance of assuming a developmental and processual perspective in studying it (Bowden, 2009; Gambetti et al., 2012). Finally, more widely shared seems to be the conceptualization of patient engagement as the incremental result of individual maturation or healthcare organizational changes (Hibbard et al., 2008; Carman et al., 2013; Graffigna et al., 2014).

### Third proposition: engagement is a self-transformative experience

Across the body of literature analyzed, engagement is described as the result of an individual's deliberate decision to modify his/her role in the organizational setting. In the case of employees, engagement is the conscious and deliberate decision of individuals to assume a more proactive role in task completion and in organizational life participation (Eldor, 2016). In the area of consumer behavior, engagement is a function of the free decision of clients to engage in a deeper, more loyal and more participative relationship with the company (Verleye et al., 2014; Kumar and Pansari, 2016). Finally, in the case of patients, engagement is related to the individual's decision to take an active and partnership position in the care journey (Coulter and Ellins, 2007; Hibbard and Cunningham, 2008).

Engagement is thus also described as the result of a sort of self-transformation and or acquisition of psychological ownership of one's role in the organizational setting. This self-transformation in the direction of a new role identity in the organization appears not only distinctive of the literature debate on engagement, but also promising for laying the bases of an engagement theory within organizational settings. In other words, the development of individuals' engagement in organizations, in the case of both “producers” or “clients” of organizational services and processes, consists in the development and assumption of the new role of “co-authors” of the organizational processes themselves.

### Fourth proposition: engagement develops within a relational context

The literature on employee engagement positions the theoretical development of this concept within the paradigm of organizational relationship theories (Macey and Schneider, 2008). Similarly, scholars involved in the debate on consumer engagement set this concept within the domain of brand relationship theories (Bowden, 2009) and within the framework of value co-creation (Vargo and Lusch, 2008). Finally, the analysis of patient engagement is located within the framework of patient-centered medicine (Bardes, 2012), which advocates the collaborative and democratic relationship between healthcare professionals and patients.

Thus, it is evident that engagement in organized settings is a purely relational concept which relates to how an individual may relate (in a more or less engaged way) with another individual, a task, or even the organization as a whole. In other words, engagement in organized settings may be defined as a psychological experience which qualifies the systemic relation that occurs within organizations at the level of symbolic and interpersonal exchanges. This ultimately relational nature of engagement may be considered another fundamental component of this concept which differentiates it from the kindred concepts of activation and empowerment. These latter concepts, in fact, appear more related to the level of power, skills and knowledge of single individuals in performing their activities than to how individuals change their state of mind and self-identity in relation to an organizational goal or setting. As suggested by Fumagalli et al. (2015) in regard to patient engagement (but this theorization seems exportable to the other settings of analysis), engagement may be considered the motivational level and enabler of empowerment and activation.

### Fifth proposition: engagement is a systemic phenomenon

On the basis of the previous arguments, and due to the assumption of the multi-componential and complex nature of the engagement phenomenon evidenced by the literature analyzed, we assume that an oversimplifying approach to the analysis (and promotion) of engagement in organized settings is not advisable. The issue of “disengagement”—both of employees and end clients—requires a holistic and systemic approach to investigate and solve its underlying causes. Furthermore, due to the inner relational nature of engagement, not only intra-psychological or individual factors to improve (or hinder) engagement should be explored: rather, a multilevel approach to this concept should be preferred. From our perspective, engagement should be seen as a new paradigm in organization functioning and services delivery, oriented to supporting cooperation and “co-authorship” among all the different actors involved in such processes, both as producers and end clients. This means fostering the emergence of an organizational ecosystem of engagement including synergic actions addressing the multi-layer factors affecting the engagement of both employees and of consumers.

## Conclusions and study limits

Engagement is today considered a promising means to sustain the transformation and survival of organizations in the current competitive scenario. Promoting a more participative and co-authorial involvement of human resources and end clients in organizations appears to be an ethical and pragmatic priority for both scholars and practitioners. However, promoting the effective engagement of both employees and consumers (and patients in the care of healthcare organizations) should be seen as a long and complex process, which needs continuous fine-tuning between evidence from scientific research and clinical practices. Employees' and clients' voices in this process should also be taken into greater consideration. Actualization of the organizational engagement imperative, in other words, requires a deep cultural change in how organizational processes and services are designed and delivered. At present, although a large body of evidence has been produced in the literature, the debate on engagement still appears to be in its infancy and needs further investigation to reach better consensus and theoretical foundation. Furthermore, the debate on engagement in diverse organizational settings and related to different actors involved in such settings (such as employees, consumers and patients) has never been bridged? With the consequent loss of cross-fertilization and transmission of insights and useful knowledge.

In this scenario, some crucial questions oriented our investigation and the preliminary results reported in this paper: *can we consider engagement as a unique phenomenon across organizational settings? Are the engagements of employees, consumers and patients diverse or similar in their inner psychological nature? Is it possible to overcome theoretical boundaries to open a cross-disciplinary debate on engagement in organizational settings?*

Although our concept analysis of the literature was preliminary and limited, some first promising insights emerged in regard to interesting similarities among these three variants of the engagement phenomenon: employee, consumer, and patient engagement. However, a more systematic and broad analysis of the literature should be conducted. Our analysis was limited to only three forms of engagement, whereas other concepts such as student engagement, stakeholder engagement, and organizational engagement should be included in the analysis to furnish further insights into the nature of engagement in organized settings. Moreover, not only should the previous literature on engagement be taken into account in order to propose a shared foundation of engagement in organizations, but new transdisciplinary research should be conducted to explore this phenomenon better and to investigate how engagement may change across organizational settings and cultural domains.

Our preliminary analysis was only aimed at proposing an agenda for future conjoint research and investigation in the area of engagement in organized settings. We truly believe that only a multi-disciplinary and multi-stakeholder consensus can transform engagement from a “buzzword” to a shared corpus of knowledge and best practices!

## Author contributions

GG, ideated and conducted the analysis and prepared the manuscript.

### Conflict of interest statement

The author declares that the research was conducted in the absence of any commercial or financial relationships that could be construed as a potential conflict of interest.
